# A Longitudinal Study of NADC34-Like Strains in an Intensive Farm Unravels Divergent Evolution

**DOI:** 10.1155/2023/3869145

**Published:** 2023-11-16

**Authors:** Sijia Xu, Jing Liu, Jiabao Xing, Han Gao, Dihua Zhu, Zhiying Xu, Jianhao Zhong, Yue Li, Xiaopeng Gao, Qiyuan Kuang, Guihong Zhang, Heng Wang, Yankuo Sun

**Affiliations:** ^1^Key Laboratory of Zoonosis Prevention and Control of Guangdong Province, College of Veterinary Medicine, South China Agricultural University, Guangzhou, China; ^2^National Engineering Research Center for Breeding Swine Industry, South China Agricultural University, Guangzhou, China; ^3^Maoming Branch, Guangdong Laboratory for Lingnan Modern Agriculture, Maoming 525000, China

## Abstract

NADC34-like porcine reproductive and respiratory virus (PRRSV) has had a significant impact on the pig industry, particularly in China. However, the evolutionary characteristics and pathogenicity of NADC34-like PRRSV strains within intensive farming systems are not well understood, particularly regarding the biological characteristic variation of successive outbreaks on a farm. In this study, we conducted continuous surveillance in an intensive farm that experienced a PRRSV outbreak. Two PRRSV strains, GDHZ109/2020 and GDYS162/2022, were isolated and fully sequenced from the same swine farm in Guangdong Province in 2020 and 2022, respectively. Evolutionary analysis based on the ORF5 gene revealed that both strains clustered with NADC34-like strains and shared 96.5% homology. Analysis of the full-length genome and NSP2 gene classified the strains into lineage 1.8, represented by the NADC30 strain. Recombination analysis suggested complex recombination patterns for both strains, involving NADC30-like, NADC34-like, and JXA1-like strains. Although many recombinant regions were nearly identical, there were differences observed in the NSP5–NSP7 region. Pathogenicity experiments conducted on piglets demonstrated that GDHZ109/2020 exhibited higher pathogenicity compared to GDYS162/2022. Piglets in the GDHZ109/2020 infected group had more severe clinical signs and higher mortality. Moreover, analysis of respiratory microbial diversity indicated a significant increase in the abundance of potentially pathogenic microbiota, such as *Klebsiella* and *Erysipelothrix* contributing to the respiratory tract of PRRSV-infected piglets, suggesting secondary infections due to differences in immune defense. These findings highlight the importance of NADC34-like recombinant strains' evolution during the farm's epidemic period, which may have contributed to changes in pathogenicity. This study improves our understanding of the current prevalence of PRRSV and provides novel insights into the prevention and control of PRRSV.

## 1. Background

Porcine reproductive and respiratory syndrome (PRRS) is a disease in pigs that causes respiratory and reproductive problems. It is caused by the porcine reproductive and respiratory syndrome virus (PRRSV) and has resulted in significant economic losses for the global pig industry [1, 2]. The PRRSV genome belongs to the *Betaarterivirus* genus of the *Arteriviridae* family in the *Nidovirales* order, with a length of 15–16 kb [3, 4]. The RNA-dependent RNA replicase lacks a genome correction mechanism during replication, which makes it susceptible to genetic recombination and mutation [5]. This results in the complexity and diversity of current PRRSV strains. PRRSV strains are currently divided into two major groups, genotype I and genotype II, with a genetic similarity ranging from 50% to 60% genotype II strains, which have become a global epidemic, have been identified based on ORF5 genotyping [6–8]. There are nine lineage of genotype II strains that have been identified and distributed in various regions of the world [9].

PRRSV has two prevalent genotypes in China: genotype I and genotype II. Genotype II has the greatest impact on the Chinese pig industry [10]. There are currently four prevalent clades of genotype II strains in China: Lineage 1, Lineage 8.7, Lineage 5.1, and Lineage 3 [11, 12]. Since 2013, the Lineage 1 clade, represented by the NADC30 strain, has spread and become predominant in various regions of China, posing a significant threat to the pig industry [13, 14]. Studies have shown that sublineage 1 strains, including Lineage 1.1, Lineage 1.5, and Lineage 1.6, have emerged in China [15–17]. The sublineage 1.5 clade, represented by the NADC34 strain, is of particular concern in China [18, 19]. There is evidence that this strain was previously reported to be more pathogenic than the NADC30 strain, causing dramatic abortion storms in sows and high mortality rates in piglets in the United States in 2014 [20, 21]. It also caused significant economic losses to the Peru pig industry from 2015 to 2017 [22]. Currently, the NADC34-like strain is endemic and spreading in different regions of China, but relatively little research has been done on this strain. Current studies have shown that there are differences in the pathogenicity and clinical symptoms of different PRRSV strains. The changing characteristics of NADC34-like strains and their development trend in China need to be studied.

The balance and relationships of the microbiota in health and disease are complex and not well understood. However, there is growing evidence that the diversity and composition of the microbiome play an important role in the regulation, elimination, and enhancement of infectious diseases. An emerging area of research that has recently emerged is exploring the important role of the microbiota in response to extragastrointestinal infections, such as respiratory and systemic infections. Results from respiratory tract infections of influenza virus, *Streptococcus pneumoniae*, *Staphylococcus aureus*, and *Klebsiella pneumoniae* have all been shown to be associated with enteric microbial composition. Disease infection and microbiota relationships in pigs have also been studied, including studies on *Mycoplasma hyopneumoniae* and coinfection with PRRSV and PCV2. PRRSV has been endemic for many years, but we failed to produce a vaccine that protects broadly or develop programs that can long-term eliminate the virus in pig-intensive areas. Therefore, it is necessary to conduct research on the respiratory microbiota to provide alternative solutions for the prevention and control of PRRSV.

In this study, we monitored NADC34-like strains in the same swine-raising farm region in South China in 2020 and 2022, nominated GDHZ109/2020 and GDYS162/2022, respectively. After a background investigation, the outbreak of the NADC34-like strain in this region in 2020 resulted in 35% of severe miscarriage in sows and a 30% culling rate of piglets on farms. However, in 2022, we observed a milder virulence of NADC34-like strains in this region, with a significant reduction of 10% in sow abortion and 5% in piglet culling rates. In response to this interesting observation, we carried out isolation, genomic sequencing, and analysis of the novel strains, as well as pathogenicity determination. To investigate the effect of these two strains on the respiratory microbiota balance of piglets, we determined the changes in the respiratory microbiota of infected pigs along with the pathogenicity by Metagenomics techniques to provide a reference for the prevention and control of PRRSV in China.

## 2. Materials and Methods

### 2.1. Ethics Statement

Animal-related research procedures in this study followed the guidelines set by the South China Agricultural University Animal Care and Use Committee for sampling. The animal experimentation was conducted supervised by the Animal Ethics Committee of South China Agricultural University (No. 2022c065).

### 2.2. Sample Collection and Virus Isolation

A total of 10 serum samples were collected from a swine-raising farm without PRRSV vaccination history in Guangdong Province, Southern China, where piglets exhibited dyspnea and abortion in 2020 and 2022, respectively. Sample processing was performed as previously described [23]. RNA was extracted from serum samples using the quick RNA extraction kit (RNAfast200, Fastgen, Shanghai, China), and PRRSV was detected by RT-PCR (TaKaRa Co., Dalian, China). Subsequently, we also screened these serum samples for ASFV, CSFV, PCV2, and PRV pathogens and the results were negative. Porcine alveolar macrophages (PAMs) were obtained from 4-week-old SPF piglets. The PRRSV-positive serum samples were filtered through a 0.22 *µ*m syringe filter (Millipore, Billerica, MA, USA), inoculated onto PAMs and Marc-145 cells for virus isolation. Nucleic acid and IFA testing were conducted for more than three consecutive generations to observe successful isolation of the virus. Two PRRSV strains were isolated and identified by IFA testing and next-generation sequencing (NGS) after culturing on PAMs to the third passage. After that, the proliferative ability of the two isolates was tested.

### 2.3. Immunofluorescence Assay

Viral supernatant was harvested after 3 days of incubation and stored as viral stocks at −80°C. The isolated PRRSV strains were inoculated into PAMs in 96-well plates. After 1 hr of incubation, cells were washed three times with PBS, fixed with cold acetone, and blocked with 5% bovine serum albumin. Monoclonal antibodies against PRRSV N protein were added to the cells and incubated for 1 hr. Subsequently, cells were washed three times with PBS and incubated with goat antimouse IgG secondary antibody for 1 hr. Finally, cells were washed three times with PBS and examined under a fluorescence microscope in PBS. To evaluate the growth kinetics of the two isolates, PAMs were infected with a multiplicity of infection of 0.1.

### 2.4. Next-Generation Sequencing

Genomic RNA of two strains was extracted from clinical specimens using the previously described method. Full-length sequences of PRRSV genomic RNA were obtained using MGI-SEQ200, PE100 (MGI, Shenzhen, China) as described previously [24, 25]. In brief, viral RNA was extracted from these samples using QIAamp Viral RNA kits (Qiagen, Germany). Library preparation was performed using the Library Prep Kit for MGI (Vazyme, Nanjing, China), and the samples were sequenced on the MGI platform at BGI, Shenzhen to obtain sequencing data for NGS. Clean reads (>Q30) were de novo assembled into viral contigs using Megahit v1.2.8. The contigs were annotated using Diamond 0.9.22, and complete PRRSV genomes were successfully recovered.

### 2.5. Evolutionary Analysis

Representative strains of PRRSV were obtained from GenBank, and multiple sequence alignments were computed using MAFFT [26]. We used ORF5, NSP2 genes, and complete genomes of reference strains to construct a maximum-likelihood tree (ML tree) with our sequences. We used the best-fit nucleotide substitution model system, which automatically matches with a bootstrap of 1,000 replicates, using IQ-TREE software [27]. The results were further visualized using Figtree v1.4.4 (http://tree.bio.ed.ac.uk/software/figtree/). We used the ggtree package in R v4.1.2 to analyze the deduced amino acid (aa) sequence of the NSP2 gene of each strain based on the results of the ML tree of the NSP2 gene [28].

### 2.6. Recombination Analysis

To evaluate the recombinant events of the GDHZ109/2020 and GDYS162/2022 strains, we only considered recombination events that were supported by at least three of seven recombination detection algorithms (RDP, GENECONV, BootScan, MaxChi, Chimaera, SiScan, and 3Seq) in the Recombination Detection Program 4 (*p*-value < 0.001). The identified recombination events were confirmed using Simplot 3.5.1, which was performed within a 200 bp window, sliding along the genome alignments with a 20 bp step size. We further confirmed all breakpoints using RT-PCR and Sanger sequencing.

### 2.7. Pathogenicity Evaluation

All SPF piglets aged 4 weeks were confirmed to be negative for PRRSV, CSFV, PRV, and PCV2 by using RT-PCR and the PRRSV Antibody Test Kit 2XR (Herd Check ELISA, IDEXX Laboratories). Two experimental groups were established: the GDHZ109/2020 challenge group and the GDYS162/2022 challenge group, each comprising five pigs that were intramuscularly and intranasally challenged with 2 mL (2 × 10^5^ TCID_50_/mL) of viral cultures at each site. In addition, three sentinel pigs were included to mimic natural infection. Piglets in the control group were intramuscularly and intranasally inoculated with 2 mL DMEM. Body temperature and clinical signs were monitored and recorded throughout the experiment. Swab and serum samples were collected at 0, 3, 7, 10, 14, and 21 days postinfection (dpi). At 21 dpi, all surviving pigs were euthanized and necropsied, and the collected tissues were used for respective assays.

### 2.8. Microbial Diversity Sequencing and Data Analysis

The deep respiratory swabs of each piglet in the GDHZ109/2020-infected group were collected for microbial profiling. Specifically, DNA from the samples was extracted using the QIAamp DNA Micro Kit (Qiagen) following the QIAamp DNA Micro Kit protocol. Then, primer pairs 338F (5ʹ-ACTCCTACGGGAGGCAGCAG-3ʹ) and 806R (5ʹ-GGACTACHVGGGTWTCTAAT-3ʹ) were used to amplify the hypervariable region V3–V4 of the bacterial 16S ribosomal RNA gene (∼291 bp) by an ABI GeneAmp® 9700 PCR thermocycler (ABI, CA, USA). Libraries were paired-end sequenced on an Illumina MiSeq PE300 platform (Illumina, San Diego, USA) according to standard protocols by Majorbio Bio-Pharm Technology Co. Ltd. (Shanghai, China). The raw data was made available in the NCBI sequence read archive database under the Bioproject: PRJNA1016119. After data processing, the UPARSE version 7.1 software was deployed to cluster all the effective tag sequences at 97% sequence similarity and to obtain operational taxonomic units (OTUs), respectively [29]. Then the absolute abundance and relative information of tags of OTUs were calculated for each sample. The bacteria were categorized according to representative sequences by a naive Bayesian model using RDP (Ribosomal Database Project) classifier (Version 2.2) based on SILVA Database [30, 31]. Subsequent statistical analyses and microbial community variation were calculated based on this file.

### 2.9. Statistical Analysis

In this study, *t*-tests and multiple comparisons were performed to compare the differences in the means of changes in rectal temperatures, antibody levels, and virus copy numbers in each group of piglets. All the data in this report was shown as the means ± SDs. Data analysis was conducted using GraphPad Prism 5 software (San Diego, CA, USA). The level of significance was set at *p* < 0.05.

## 3. Results

### 3.1. Virus Isolation and Identification

**Figure 1:**
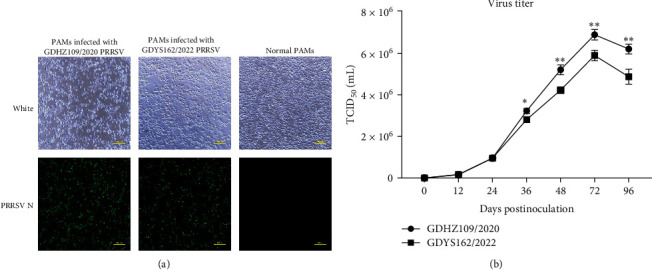
Infection and proliferation of isolated strains in PAMs. (a) Passages of GDHZ109/2020 and GDYS162/2022 in PAMs were detected at 40x magnification. (b) PAMs were infected with GDHZ109/2020 and GDYS162/2022 at an MOI of 0.1. The viral supernatants were collected at indicated time points from infected cells and titrated by IFA. All tests were performed in triplicate and repeated twice ( ^*∗*^*p* < 0.05;  ^*∗∗*^*p* < 0.01).

Two PRRSV strains were successfully isolated from serum samples suspected of infection. After three generations of passaging on PAMs and Marc-145 cells, RT-PCR and IFA showed positivity only in PAMs (Figure 1(a)). Indeed, Marc-145 cells were negative for both strains for over 10 passages, as assessed by RT-PCR, CPE, and IFA. Therefore, Marc-145 cells were considered not susceptible to GDHZ109/2020 and GDYS162/2022. To determine the replication kinetics of the two novel strains, the cell culture supernatant was quantified by titration after serial tenfold dilution and detected at different time points, such as 12, 36, 48, 72, and 96 hours postinfection (hpi). The GDYS162/2022 strains had an overall reduced growth ability compared to GDHZ109/2020 (Figure 1(b)).

### 3.2. Phylogenetic Analysis and Genomic Characteristics

**Figure 2:**
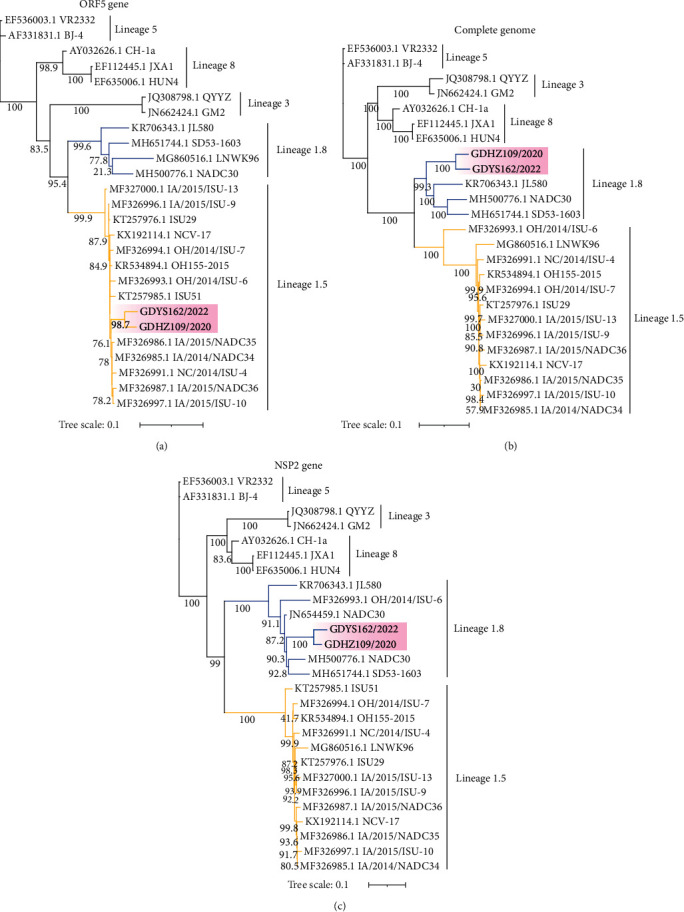
Phylogenetic tree of isolated strains. ML trees were constructed based on the nucleotide sequences of representative strains for ORF5 (a), complete genome (b), and NSP2 (c), with bootstrapping at 1,000 replicates. The isolated strains are highlighted in pink shading.

The whole genome of GDHZ109/2020 and GDYS162/2022 strains were 15,019 and 15,127 bp, shared 89.0% and 88.7% sequence identity with NADC30 (MH500776.1), respectively. The sequence similarity between the complete genome of two novel strains was 95.4%, while that between the ORF5 gene of two novel strains was 96.5%. To establish genetic relationships between two novel strains and other PRRSV isolates, phylogenetic trees based on the complete genome sequence, NSP2 gene and ORF5 gene were constructed and using PRRSV strains available in GenBank. Phylogenetic analysis demonstrated that the two strain sequences can be classified into lineage 1.8, represented by NADC30, based on the full-length genome sequence and NSP2 (Figures 2(b) and 2(c)). The popular Chinese PRRSV II strain can be divided into four lineages based on the ORF5 sequence, including NADC30-like (lineage 1), QYYZ-like (lineage 3), VR2332-like (lineage 5/5.1), and JXA1-like/CH-1a-like (lineage 8/8.7) (representing strains of each lineage). Notably, the GDHZ109/2020 and GDYS162/2022 strains were clustered into a separate branch of the phylogenetic tree based on their ORF5 genotyping, which were located on lineage 1.5, represented by NADC34-like (Figure 2(a)). These results indicated that recombination events might have occurred in the genomes of GDHZ109/2020 and GDYS162/2022 strains. Two strains in this study shared a common “111 + 1 + 19” deletion pattern compared to the NSP2 gene of the VR2332 strain, which was exactly the same pattern observed for the NADC30 strain or K07-2273 strain (*Supplementary 1*).

### 3.3. Recombination Analysis

**Figure 3:**
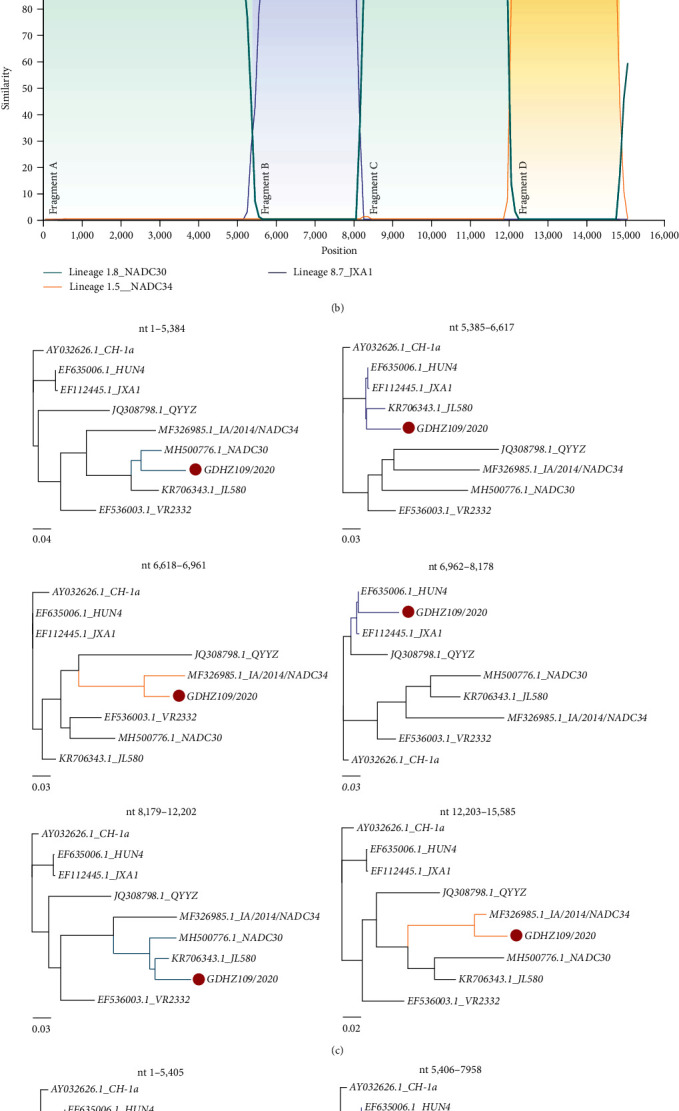
Recombination and phylogenetic analysis based on different regions of GDHZ109/2020 (a) and GDYS162/2022 (b). The likely recombination region is shaded in a different color and colored broken lines represent different lineages: green indicates NADC30-like PRRSV (Lineage 1.8), yellow indicates NADC34-like PRRSV (Lineage 1.5), and blue indicates JXA1-like PRRSV (Lineage 8.7). (c and d) Maximum likelihood phylogenetic trees inferred for the different recombinant regions. The ML tree was reconstructed using the best-fit nucleotide substitution model system. 1,000 bootstraps were evaluated to assess support values. NADC30-like PRRSV (Lineage 1.8) is indicated in green, NADC34-like PRRSV (Lineage 1.5) in yellow, and JXA1-like PRRSV (Lineage 8.7) in blue. The red dots indicate strains sequenced in this study.

Both isolates showed robust but complex recombination signals involving NADC30-like, NADC34-like, and classic HP-PRRSV strains circulating in China (Figure 3). Three recombination breakpoints were identified in the GDYS162/2022 complete sequence, located in NSP3 (5,405 nucleotides (nt)), NSP9 (7,958 nt), and ORF2a (12,195 nt), based on the similarity plot (Figure 3(b)). Based on the recombinant breakpoints, the GDYS162/2022 complete sequence was divided into four regions, with NADC30 as the major parent and NADC34 and HP-PRRSV as minor parents. Phylogenetic analysis indicated that region A (5ʹUTR-NSP3, 1–5,405 nt) and region C (NSP9-ORF2a, 7,959–12,195 nt) were clustered with NADC30 (MH500776.1) (Lineage 1.8), region B (NSP3–NSP9, 5,406–7,958–nt) was closely clustered with the JXA1 strain (Lineage 8), and region D (ORF2a-3UTR, 12,196–15,588 nt) was closely associated with NADC34 strain (MF326985.1) (Lineage 1.5), according to the phylogenetic analysis.

However, the GDHZ109/2020 strain exhibited a slightly different recombination pattern than the GDYS162/2022 strain (Figure 3(a)). The recombination breakpoints divided the complete genome of the GDHZ109/2020 strain into six regions, including region A (1–5,384 nt), region B (5,385–6,617 nt), region C (6,618–6,961 nt), region D (6,962–8,178 nt), region E (8,179–12,202 nt), and region F (12,203–15,588 nt), with NADC30 as the major parent and NADC34 and HP-PRRSV as minor parents. Region A and region E were closely related to NADC30-like strain (Lineage 1), whereas region B and region D were clustered with JXA1-like strains (Lineage 8). Unlike the GDYS162/2022 strain, GDHZ109/2020 recombined twice with NADC34-like strain, involving region C and region F as parent strains. These results suggest that the GDHZ109/2020 and GDYS162/2022 strains likely originated from multiple recombination events among NADC30, JXA1, and NADC34-like strains, with GDHZ109/2020 exhibiting a more complex recombination event with the NADC34 strain.

### 3.4. Pathogenicity Analysis

**Figure 4:**
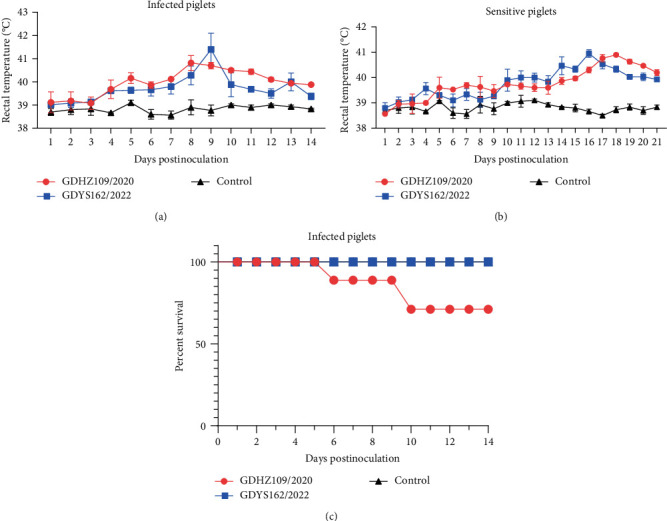
Rectal temperature and survival rate during the challenge study. (a) Rectal temperatures of piglets inoculated with GDHZ109/2020, GDYS162/2022, and control group are shown as mean ± SD (error bars) temperatures (°C). The fever cut-off value was set at 40.0°C. (b) Rectal temperature of sensitive piglets in various groups. (c) Survival and mortality curves for inoculated piglets.

The animal experiment results indicated that piglets inoculated with GDHZ109/2020 displayed evident clinical signs, such as respiratory distress, anorexia, and coughing, from 5 dpi, while the GDYS162/2022 group began to exhibit obvious clinical signs from 7 dpi. The rectal temperature of infected piglets showed a febrile response (40°C) at 5 dpi, and the highest rectal temperature of the GDHZ109/2020 challenge group and the GDYS162/2022 challenge group were observed at 8 and 9 dpi, respectively (Figure 4(a)). The sensitive piglets exhibited a delayed onset of febrile response, and the highest temperature was observed at 16 and 18 dpi, respectively (Figure 4(b)). By contrast, the piglets in the control group showed no evident clinical signs and maintained normal temperature throughout the study. Two infected piglets of the GDHZ109/2020 challenge group were euthanized due to their moribund conditions at 6 and 9 dpi, respectively (Figure 4(c)). The remaining infected piglets in the challenge group survived and were euthanized at 14 dpi, whereas all of the sensitive piglets were euthanized at the end of the study.

### 3.5. Viremia, Viral Shedding, and Tissue Viral Loads Detection

**Figure 5:**
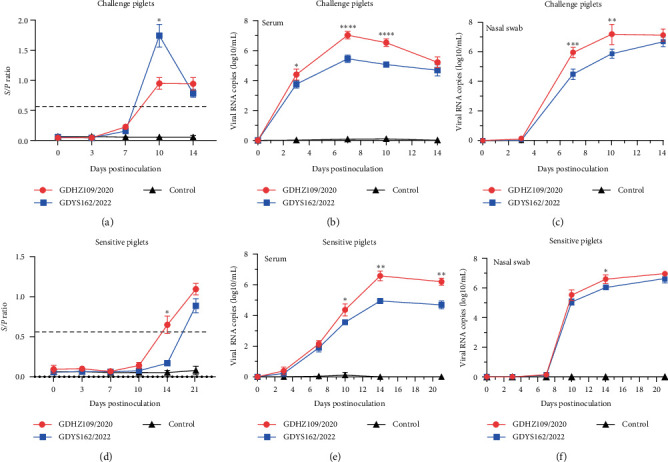
Viremia and antibody levels of pigs during the challenge experiment. (a–c) Represent the antibody levels, viral load in serum samples, and nasal swabs for challenged piglets. (d–f) Represent the antibody levels, viral load in serum samples, and nasal swabs for sensitive piglets. Serum was tested using the IDEXX PRRS X3 ELISA kit, with positivity at an *S*/*P* value > 0.6 ( ^*∗*^*p* < 0.05;  ^*∗∗*^*p* < 0.01;  ^*∗∗∗*^*p* < 0.001).

Antibody levels and viral loads were detected in serum samples collected from individual piglets at 0, 3, 7, 10, 14, and 21 dpi. The antibody level of the GDHZ109/2020-infected group peaked at 10 dpi and decreased thereafter (Figure 5(a)). At 10 dpi, all infected piglets in the challenge group had PRRSV-specific positive antibodies (according to the instructions, *S*/*P* > 0.6 is positive). Serologically positive sensitive piglets had a later onset of presentation at 14 dpi and kept increasing until the end of the study (Figure 5(d)). Piglets in the control group were serologically negative during the study period. Second, the viral load test results indicated that both the GDHZ109/2020-infected group and GDYS162/2022-infected group exhibited a gradual increase in viremia level from 0 to 7 dpi and reached the highest viremia level at 7 dpi (Figure 5(b)). The average level of the peak at 7 dpi reached 2 × 10^8^ copies/mL. After that, the viral load in the serum of the two challenge groups gradually decreased until the end of the study. For the sensitive piglets, the highest viral load in serum appeared at 10 dpi, reaching a peak viral load with an average level of 6 × 10^7^ copies/mL (Figure 5(e)). As expected, there was no viremia detected in the piglets in the control group.

In addition, nasal swabs were collected to determine virus shedding in the respiratory route. Virus load was detectable in all five nasal swabs in the GDHZ109/2020-infected group at 7 dpi, whereas three of five swabs in the GDYS162/2022-infected group had virus load at the same time (Figure 5(c)). For sensitive piglets, virus shedding in nasal swabs began at 10 dpi and gradually increased until the end of the study (Figure 5(f)). By contrast, no virus shedding was detected in the control group.

### 3.6. Macroscopic and Histopathological Lesions

**Figure 6:**
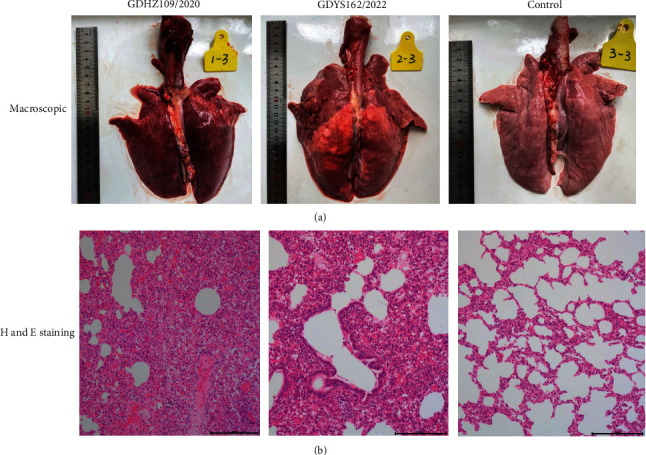
Pathological changes in lungs during the challenge experiment. (a) Macroscopic lesions in the lungs of piglets in each group. (b) Representative images of lung tissues stained with H&E.

To further verify the pathological changes in the organs of infected piglets, tissues were collected at necropsy, and formalin-fixed tissues were made into paraffin sections and stained with hematoxylin and eosin. The challenge group showed typical lesions of PRRSV, such as pulmonary consolidation and ecchymosis (Figure 6). Individual piglets also developed emphysema. In addition, the lymph nodes in the mediastinal and inguinal regions showed enlargement and hyperplasia, and some piglets had congestion and hemorrhage in the hilar lymph nodes. Furthermore, some piglets had pleural effusion, which is the accumulation of fluid in the pleural space. The pathological changes in the lungs of sensitive piglets are similar to those in infected piglets.

Histopathology revealed that pulmonary lesions of the challenge group presented as interstitial pneumonia, showing obvious widening of the alveolar septum and infiltration of a large number of inflammatory cells. The pulmonary lesions of individual piglets presented as multifocal hemorrhages and cell necrosis. In contrast, macroscopic and histopathological lesions of the control group showed normal morphology and staining of all tissues, and the organs had no significant lung pathology.

### 3.7. Respiratory Microbial Diversity

**Figure 7:**
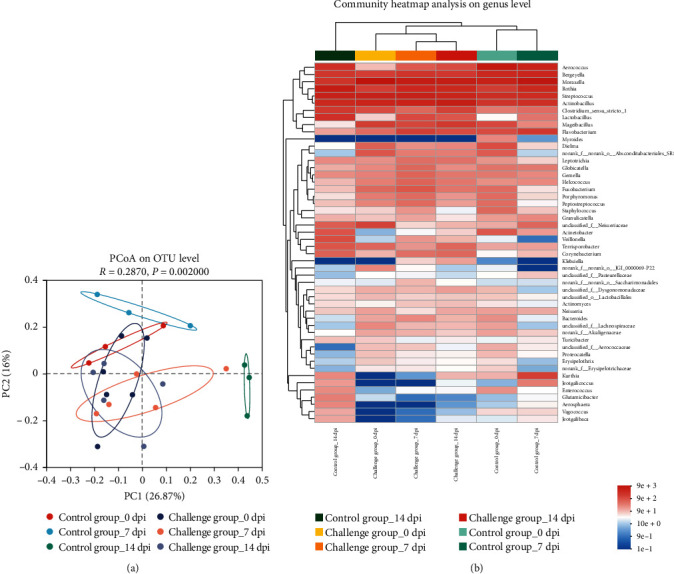
Differences in respiratory microbiota of piglets in GDHZ109/2020 group and control group. (a) PCoA ordination plots for beta diversity (Bray–Curtis metric) for each group's samples. Each dot represents a sample's microbial community, with different groups represented by different colors. The first axis explains 26.87% of the variability, and the second axis explains 16% of the variability in the data of samples (*p* < 0.05). (b) Genera abundance analyses in the challenge and control groups.

Respiratory microbial diversity was analyzed to explore the correlation between respiratory microbiota and viral pathogenicity. Bray–Curtis dissimilarity measure was used to quantify beta diversity. The results indicated that the respiratory microbiota of piglets in the control group changed over time, whereas that in the challenge group tended to stabilize during the study (Figure 7(a)). Moreover, at 0 dpi, there was no significant difference between the experimental and control groups, whereas at 7 and 14 dpi, the difference between the two groups was significant (*p* < 0.05) (*Supplementary 2*). The species abundance at the genus level was quantified for each sample, and the taxonomic heatmap depicted the composition of respiratory communities in each group of piglets (Figure 7(b)). As shown, the abundance of Bacteroides, *Lachnospiraceae* in the respiratory tract of infected piglets increased while the abundance of *Enterococcus* decreased compared to the control group. Importantly, the abundance of potentially pathogenic microbiota, such as *Klebsiella* and *Erysipelothrix*, in the respiratory tract of piglets significantly increased after PRRSV infection. Furthermore, the prediction of respiratory microbiota function revealed that some function abundance changed in the challenge group compared to the control group (*Supplementary 2*).

## 4. Discussions

PRRSV has resulted in huge economic losses for the Chinese swine industry since it was first discovered in China in 1996 [10]. Despite the widespread use of vaccines on farms, PRRS in swine herds remains poorly controlled. Since 2013, a highly virulent (RFLP) 1-7-4 lineage strain of PRRSV has emerged, resulting in severe abortions in sows and high piglet mortality. In 2013–2014, Van Geelen et al. [21] described several pathogenically distinct PRRSV 1-7-4 lineage isolates in different states of the United States. Among these isolated strains, IA/2014/NADC34 (NADC34) PRRSV has been reported to be associated with sow abortion storms and high piglet mortality in at least five states in the United States since 2016 [21]. In 2017, a strain of 1-7-4 lineage PRRSV was isolated in China, which had the highest genomic similarity with IA/2014/NADC34 (NADC34), named NADC34-like PRRSV [18]. Since then, NADC34-like PRRSV has spread to many provinces in China [32, 33]. What's more, NADC34-like PRRSV underwent a complex recombination with native Chinese strains. The two emerging strains are also indicative of this phenomenon in our study. In China, the coexistence of different lineages of PRRSV in the field, coupled with immune pressure, may exacerbate the evolution of PRRSV, making PRRS prevention and control more difficult.

PRRSV is prone to genetic variation. Two PRRSV strains, GDHZ109/2020 and GDYS162/2022, were isolated from the same swine farm in South China in 2020 and 2022. The genetic evolution and recombinant analyses suggested that both isolates may represent significant recombination events. Although the recombinant patterns of the two strains were similar, there were also some differences. Therefore, there is no direct evidence that GDYS162/2022 evolved from GDHZ109/2020. Specifically, the GDYS162/2022 strain had the highest similarity to the NADC34-like strain in the NSP5–NSP7 segment, while GDHZ109/2020 was most similar to JXA1-like strains in this segment. The detection rate of NADC34-like strains in swine farms has increased in recent years. Recombinant strains produced by NADC34-like and other strains have been reported previously, including recombinant strains produced between two lineages (lineage 1.8 and 1.5, lineage 1.5 and 3) and between three lineages (lineage 1.8, 1.5, and 8.7) [34–37]. Importantly, multiple studies have shown that different recombination patterns of PRRSV strains can result in differences in their pathogenicity or virulence [13, 38–40].

PRRSV NADC34-like strains exhibit varying levels of virulence and pathogenicity. Van Geelen et al. [21] reported that piglets infected with IA/2014/NADC34, IA/2013/ISU-1, and IN/2014/ISU-5 had more severe symptoms than those infected with IA/2014/ISU-2, which did not cause fever and had little effect on piglet growth. Furthermore, the primary epidemic strains differ among Chinese provinces, and abortion rates, mortality, and recombination breakpoints also vary among NADC34-like strains and their recombinants. For instance, the HLJDZD32-1901 strain isolated in Heilongjiang Province caused only mild clinical symptoms and had no significant impact on piglet growth, whereas the JS2021NADC34 strain isolated from Jiangsu Province resulted in piglets with persistent fever, weight loss, interstitial pneumonia, and acute bleeding, leading to high morbidity and mortality, indicating high pathogenicity [41, 42]. Thus, NADC34-like strains, whether previously isolated in the United States or subsequently detected in China, may display varying levels of pathogenicity to piglets under experimental conditions. Previous experimental results also demonstrated that NSP9 and NSP10 contribute to the lethal virulence of HP-PRRSV on piglets [43]. In this study, the ORF1a of the GDHZ109/2020 strain exhibited the highest similarity with the JXA1 strain, and its animal results also showed high pathogenicity similar to HP-PRRSV, suggesting that the genomic components of HP-PRRSVs may enhance the virulence of GDHZ109/2020 isolates. Interestingly, in the GDYS162/2022 isolate, NSP4–NSP5 and NSP7–NSP8 had the highest similarity with the JXA1-like strain, but its NSP6 segment was more similar to the NADC34-like strain. Consequently, its infection experiments demonstrated weak pathogenicity. The above results showed that the same farm strain mutated during the epidemic process in this region, and its pathogenicity was relatively weakened, whether the reduced pathogenicity of this early emerging strain is related to the adaptive evolution of the strain needs further investigation.

A strong correlation between changes in respiratory microbiota and the pathogenicity of PRRSV strains [44–47]. 16S rRNA compositional sequencing enables the estimation of the relative abundance of various bacterial groups [48]. The detailed analysis of differences in abundance aimed to identify the challenging bacterial groups involved in the observed shift in the microbiome after PRRSV infection. In this study, the abundance of *Erysipelas* and *Klebsiella* in the respiratory tract of infected piglets was increased. *Klebsiella* infection of the respiratory tract of piglets leads to respiratory diseases such as pneumonia. Beta diversity analysis investigates the similarities or differences in community composition between samples from different subgroups by comparing species diversity across habitats or microbial communities. In our analysis, the respiratory microbiota of infected piglets tended to stabilize. It can be presumed that the increased abundance of microbiota with a synergistic effect after PRRSV infection was the cause. Previous studies have suggested that correlation analysis performed on respiratory samples from immune-deficient individuals with infections provided insight into the synergistic relationship between respiratory pathogens [49]. The infection of PRRSV causes changes in animal organism functions, which may lead to secondary infections by other pathogens.

## 5. Conclusion

In summary, we describe and characterize two novel recombinant NADC34-like PRRSV strains isolated from the same swine farm in Guangdong Province in 2020 and 2022, respectively. Despite the many similarities between these two strains, they display varying pathogenicity. This highlights the necessity for ongoing whole-genome surveillance of regional epidemic strains, as they may undergo genetic variations at different times in the field. These findings provide a better understanding of the current prevalence of PRRSV and offer a reference for the prevention and control of PRRSV in the pig industry.

## Data Availability

The 16s raw sequencing files generated in this study have been deposited in the SRA database under accession code PRJNA1016119. The raw viral sequences generated in this study have been uploaded in the CNGB database under project CNP0004883. Other data that support the findings of this study are available from the corresponding author upon reasonable request.

## Abbreviations

PRRSV: Porcine reproductive and respiratory syndrome virus
RdRp: RNA-dependent RNA replicase
NSP: Nonstructural protein
ORF: Open reading frame
PAMs: Porcine alveolar macrophages
MOI: Multiplicity of infection
IFA: Immunofluorescence assay
OTU: Operational taxonomic units
RFLP: Restriction fragment length polymorphism
dpi: Days postinfection
hpi: Hours postinfection
CPE: Cytopathic effect
NGS: Next-generation sequencing.

## Supplementary Materials

Supplementary 1 Amino acid sequence alignment of nsp2 from GDHZ109/2020, GDYS162/2022, and other representative PRRSV strains.

Supplementary 2 Beta diversity analysis at various time points and functional prediction analysis results.

Supplementary 3 The similarity between the two strains in this study and reference strain sequences.
