# Supplementary material for: A Longitudinal Study of NADC34-Like Strains in an Intensive Farm Unravels Divergent Evolution
Source: Transbound Emerg Dis. 2023 Nov 16;2023:3869145. doi: 10.1155/2023/3869145 (PMC12016764; doi:10.1155/2023/3869145)

**supplementary materials**

**Figure S1 Amino acid sequence alignment of nsp2 from GDHZ109/2020, GDYS162/2022, and other representative PRRSV strains.**

The isolated strains are highlighted by red dots.


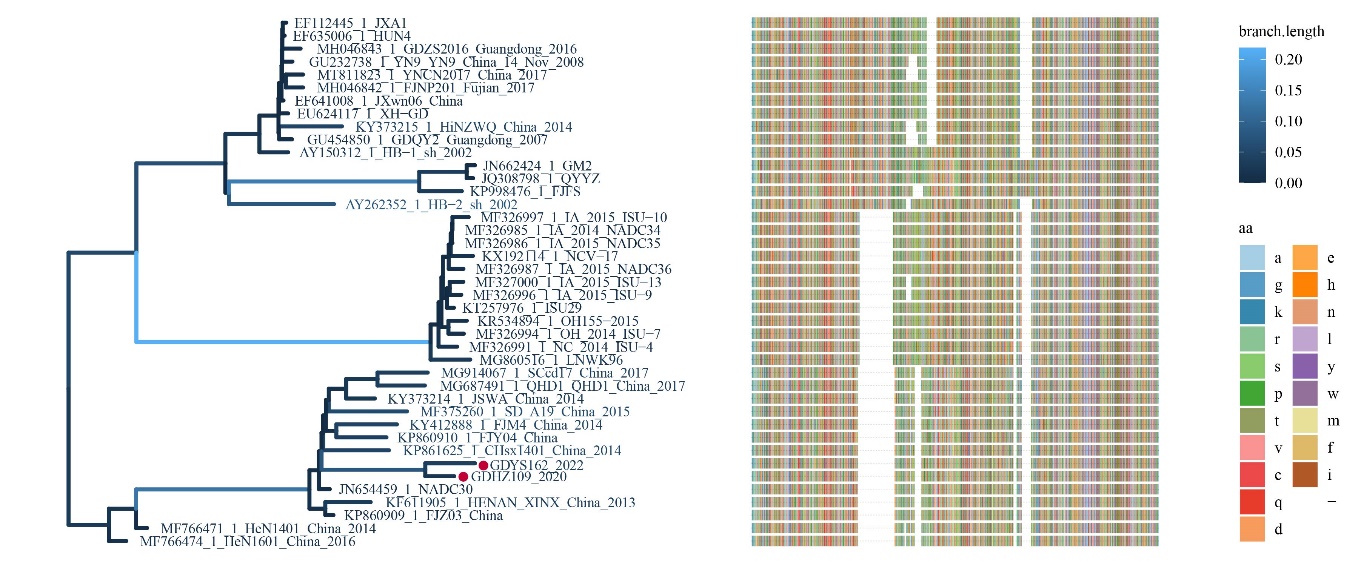


**Figure S2 Beta diversity analysis at various time points and functional prediction analysis results.**

(a) Beta diversity analysis in experimental and control groups at 0dpi, 7dpi, and 14dpi, respectively (p < 0.05). (b) The Clusters of Orthologous Groups (COG) functional analysis of microbial communities in samples was predicted from amplicon sequencing results by PICRUSt 1.1.0.


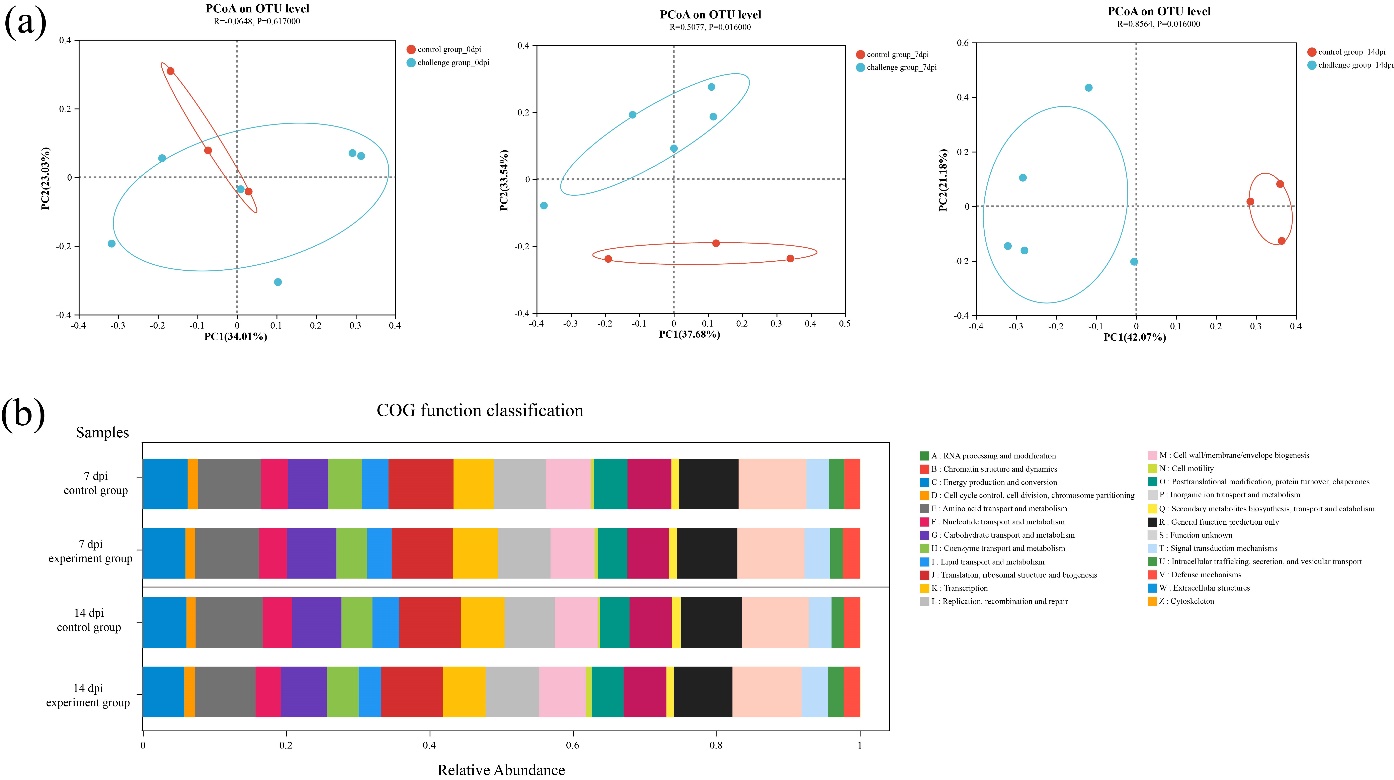

Supplement: Supplementary 1 — Amino acid sequence alignment of nsp2 from GDHZ109/2020, GDYS162/2022, and other representative PRRSV strains. [file 3869145.f1.docx]
