# Supplementary material for: A Longitudinal Study of NADC34-Like Strains in an Intensive Farm Unravels Divergent Evolution
Source: Transbound Emerg Dis. 2023 Nov 16;2023:3869145. doi: 10.1155/2023/3869145 (PMC12016764; doi:10.1155/2023/3869145)
Supplement: Supplementary 2 — Beta diversity analysis at various time points and functional prediction analysis results. [file 3869145.f2.pdf]

ORF5 gene

Pair Distances of Untitled ClustalW (Weighted)

Percent Similarity in upper triangle
Percent Divergence in lower triangle

	CH-1a	GM2	HUN4	NADC34	JXA1	JL580	ISU29	ISU51	NCV-17	NADC35	NADC36	LNWK96	SD53-1603	NADC30	QYYZ	VR2332	GDHZ109	GDYS162	
CH-1a	***	85.4	95.5	87.9	95.4	87.4	87.7	87.7	87.2	87.6	87.6	86.1	87.2	85.9	85.4	91.7	86.2	86.4	CH-1a
GM2		***	83.6	84.4	83.4	85.2	84.6	84.2	84.4	84.1	83.7	82.6	84.2	83.6	99	84.1	84.2	83.9	GM2
HUN4			***	87.2	99.8	85.6	87.1	87.1	86.6	87.2	87.2	84.7	86.6	84.7	83.6	89.1	85.6	85.9	HUN4
NADC34				***	87.1	88.1	99.3	98.8	99	99.7	99.3	86.9	88.6	87.2	84.4	88.1	96.2	96	NADC34
JXA1					***	85.4	86.9	86.9	86.4	87.1	87.1	84.6	86.4	84.6	83.6	88.9	85.4	85.7	JXA1
JL580						***	88.1	88.2	87.7	87.9	87.4	91.7	92.4	92.5	85.6	86.7	86.1	86.2	JL580
ISU29							***	98.8	99	99	98.7	86.9	88.6	87.2	84.6	87.9	95.9	95.7	ISU29
ISU51								***	98.5	98.5	98.2	86.7	88.7	87.4	84.2	87.9	95.4	95.2	ISU51
NCV-17									***	98.7	98.3	86.6	87.9	86.6	84.4	87.4	95.5	95.4	NCV-17
NADC35										***	99	86.6	88.2	86.9	84.1	87.9	95.9	95.7	NADC35
NADC36											***	86.2	88.2	86.6	83.7	87.7	95.5	95.4	NADC36
LNWK96												***	91.4	91.7	82.6	84.1	85.9	86.6	LNWK96
SD53-1603													***	94.4	84.6	86.9	86.6	86.6	SD53-1603
NADC30														***	83.9	85.6	85.7	85.4	NADC30
QYYZ															***	84.1	84.6	83.9	QYYZ
VR2332																***	86.2	86.1	VR2332
GDHZ109																	***	96.5	GDHZ109
GDYS162																		***	GDYS162
	CH-1a	GM2	HUN4	NADC34	JXA1	JL580	ISU29	ISU51	NCV-17	NADC35	NADC36	LNWK96	SD53-1603	NADC30	QYYZ	VR2332	GDHZ109	GDYS162	

Complete genome

Pair Distances of Untitled ClustalW (Weighted)

Percent Similarity in upper triangle
Percent Divergence in lower triangle

	BJ-4	VR2332	CH-1a	HUN4	JXA1	GM2	QYYZ	NADC34	LNWK96	JL580	SD53-160	NADC30	GDYS162	GDHZ109	
BJ-4	***	97.4	91	88.8	88.7	87.5	85.1	82.6	82.2	83.8	82.8	82.4	81.7	81.9	BJ-4
VR2332		***	89.8	87.5	87.5	86.2	84.2	81.9	81.5	83.1	82.1	81.6	80.7	81.2	VR2332
CH-1a			***	94.8	94.6	88	88.1	82.9	82.5	84.9	82.3	82.4	82.2	82.4	CH-1a
HUN4				***	99.6	86.6	86.8	82	81.5	84.9	81.1	81.1	81.7	81.9	HUN4
JXA1					***	86.5	86.7	82	81.5	84.8	81.1	81.2	81.6	81.9	JXA1
GM2						***	96.6	80.4	80.2	81.1	79.9	79.9	79.4	79.3	GM2
QYYZ							***	80.3	80.1	80.9	79.6	79.7	79.4	79.5	QYYZ
NADC34								***	96.2	84.9	85.4	85.2	85.4	86.2	NADC34
LNWK96									***	84.7	85.4	85.2	84.6	85.2	LNWK96
JL580										***	90.6	91	89.2	89.4	JL580
SD53-1603											***	93.7	88.6	88.9	SD53-1603
NADC30												***	88.7	89	NADC30
GDYS162													***	95.4	GDYS162
GDHZ109														***	GDHZ109
	BJ-4	VR2332	CH-1a	HUN4	JXA1	GM2	QYYZ	NADC34	LNWK96	JL580	SD53-160	NADC30	GDYS162	GDHZ109	
