# Supplementary material for: A Longitudinal Study of NADC34-Like Strains in an Intensive Farm Unravels Divergent Evolution
Source: Transbound Emerg Dis. 2023 Nov 16;2023:3869145. doi: 10.1155/2023/3869145 (PMC12016764; doi:10.1155/2023/3869145)
Supplement: Supplementary 3 — The similarity between the two strains in this study and reference strain sequences. [file 3869145.f3.docx]

| **ORF5 gene** |  |  |  |  |  |  |  |  |  |  |  |  |  |  |  |  |  |  |  |
| --- | --- | --- | --- | --- | --- | --- | --- | --- | --- | --- | --- | --- | --- | --- | --- | --- | --- | --- | --- |
| Pair Distances of Untitled ClustalW (Weighted) | | | |  |  |  |  |  |  |  |  |  |  |  |  |  |  |  |  |
|  |  |  |  |  |  |  |  |  |  |  |  |  |  |  |  |  |  |  |  |
|  | Percent Similarity in upper triangle | | | |  |  |  |  |  |  |  |  |  |  |  |  |  |  |  |
|  | Percent Divergence in lower triangle | | | |  |  |  |  |  |  |  |  |  |  |  |  |  |  |  |
|  |  |  |  |  |  |  |  |  |  |  |  |  |  |  |  |  |  |  |  |
|  | CH-1a | GM2 | HUN4 | NADC34 | JXA1 | JL580 | ISU29 | ISU51 | NCV-17 | NADC35 | NADC36 | LNWK96 | SD53-1603 | NADC30 | QYYZ | VR2332 | GDHZ109 | GDYS162 |  |
| CH-1a | *** | 85.4 | 95.5 | 87.9 | 95.4 | 87.4 | 87.7 | 87.7 | 87.2 | 87.6 | 87.6 | 86.1 | 87.2 | 85.9 | 85.4 | 91.7 | 86.2 | 86.4 | CH-1a |
| GM2 |  | *** | 83.6 | 84.4 | 83.4 | 85.2 | 84.6 | 84.2 | 84.4 | 84.1 | 83.7 | 82.6 | 84.2 | 83.6 | 99 | 84.1 | 84.2 | 83.9 | GM2 |
| HUN4 |  |  | *** | 87.2 | 99.8 | 85.6 | 87.1 | 87.1 | 86.6 | 87.2 | 87.2 | 84.7 | 86.6 | 84.7 | 83.6 | 89.1 | 85.6 | 85.9 | HUN4 |
| NADC34 |  |  |  | *** | 87.1 | 88.1 | 99.3 | 98.8 | 99 | 99.7 | 99.3 | 86.9 | 88.6 | 87.2 | 84.4 | 88.1 | 96.2 | 96 | NADC34 |
| JXA1 |  |  |  |  | *** | 85.4 | 86.9 | 86.9 | 86.4 | 87.1 | 87.1 | 84.6 | 86.4 | 84.6 | 83.6 | 88.9 | 85.4 | 85.7 | JXA1 |
| JL580 |  |  |  |  |  | *** | 88.1 | 88.2 | 87.7 | 87.9 | 87.4 | 91.7 | 92.4 | 92.5 | 85.6 | 86.7 | 86.1 | 86.2 | JL580 |
| ISU29 |  |  |  |  |  |  | *** | 98.8 | 99 | 99 | 98.7 | 86.9 | 88.6 | 87.2 | 84.6 | 87.9 | 95.9 | 95.7 | ISU29 |
| ISU51 |  |  |  |  |  |  |  | *** | 98.5 | 98.5 | 98.2 | 86.7 | 88.7 | 87.4 | 84.2 | 87.9 | 95.4 | 95.2 | ISU51 |
| NCV-17 |  |  |  |  |  |  |  |  | *** | 98.7 | 98.3 | 86.6 | 87.9 | 86.6 | 84.4 | 87.4 | 95.5 | 95.4 | NCV-17 |
| NADC35 |  |  |  |  |  |  |  |  |  | *** | 99 | 86.6 | 88.2 | 86.9 | 84.1 | 87.9 | 95.9 | 95.7 | NADC35 |
| NADC36 |  |  |  |  |  |  |  |  |  |  | *** | 86.2 | 88.2 | 86.6 | 83.7 | 87.7 | 95.5 | 95.4 | NADC36 |
| LNWK96 |  |  |  |  |  |  |  |  |  |  |  | *** | 91.4 | 91.7 | 82.6 | 84.1 | 85.9 | 86.6 | LNWK96 |
| SD53-1603 |  |  |  |  |  |  |  |  |  |  |  |  | *** | 94.4 | 84.6 | 86.9 | 86.6 | 86.6 | SD53-1603 |
| NADC30 |  |  |  |  |  |  |  |  |  |  |  |  |  | *** | 83.9 | 85.6 | 85.7 | 85.4 | NADC30 |
| QYYZ |  |  |  |  |  |  |  |  |  |  |  |  |  |  | *** | 84.1 | 84.6 | 83.9 | QYYZ |
| VR2332 |  |  |  |  |  |  |  |  |  |  |  |  |  |  |  | *** | 86.2 | 86.1 | VR2332 |
| GDHZ109 |  |  |  |  |  |  |  |  |  |  |  |  |  |  |  |  | *** | 96.5 | GDHZ109 |
| GDYS162 |  |  |  |  |  |  |  |  |  |  |  |  |  |  |  |  |  | *** | GDYS162 |
|  | CH-1a | GM2 | HUN4 | NADC34 | JXA1 | JL580 | ISU29 | ISU51 | NCV-17 | NADC35 | NADC36 | LNWK96 | SD53-1603 | NADC30 | QYYZ | VR2332 | GDHZ109 | GDYS162 |  |
|  |  |  |  |  |  |  |  |  |  |  |  |  |  |  |  |  |  |  |  |
|  |  |  |  |  |  |  |  |  |  |  |  |  |  |  |  |  |  |  |  |
|  |  |  |  |  |  |  |  |  |  |  |  |  |  |  |  |  |  |  |  |
|  |  |  |  |  |  |  |  |  |  |  |  |  |  |  |  |  |  |  |  |
| **Complete genome** | |  |  |  |  |  |  |  |  |  |  |  |  |  |  |  |  |  |  |
| Pair Distances of Untitled ClustalW (Weighted) | | | |  |  |  |  |  |  |  |  |  |  |  |  |  |  |  |  |
|  |  |  |  |  |  |  |  |  |  |  |  |  |  |  |  |  |  |  |  |
|  | Percent Similarity in upper triangle | | | |  |  |  |  |  |  |  |  |  |  |  |  |  |  |  |
|  | Percent Divergence in lower triangle | | | |  |  |  |  |  |  |  |  |  |  |  |  |  |  |  |
|  |  |  |  |  |  |  |  |  |  |  |  |  |  |  |  |  |  |  |  |
|  | BJ-4 | VR2332 | CH-1a | HUN4 | JXA1 | GM2 | QYYZ | NADC34 | LNWK96 | JL580 | SD53-1603 | NADC30 | GDYS162 | GDHZ109 |  |  |  |  |  |
| BJ-4 | *** | 97.4 | 91 | 88.8 | 88.7 | 87.5 | 85.1 | 82.6 | 82.2 | 83.8 | 82.8 | 82.4 | 81.7 | 81.9 | BJ-4 |  |  |  |  |
| VR2332 |  | *** | 89.8 | 87.5 | 87.5 | 86.2 | 84.2 | 81.9 | 81.5 | 83.1 | 82.1 | 81.6 | 80.7 | 81.2 | VR2332 |  |  |  |  |
| CH-1a |  |  | *** | 94.8 | 94.6 | 88 | 88.1 | 82.9 | 82.5 | 84.9 | 82.3 | 82.4 | 82.2 | 82.4 | CH-1a |  |  |  |  |
| HUN4 |  |  |  | *** | 99.6 | 86.6 | 86.8 | 82 | 81.5 | 84.9 | 81.1 | 81.1 | 81.7 | 81.9 | HUN4 |  |  |  |  |
| JXA1 |  |  |  |  | *** | 86.5 | 86.7 | 82 | 81.5 | 84.8 | 81.1 | 81.2 | 81.6 | 81.9 | JXA1 |  |  |  |  |
| GM2 |  |  |  |  |  | *** | 96.6 | 80.4 | 80.2 | 81.1 | 79.9 | 79.9 | 79.4 | 79.3 | GM2 |  |  |  |  |
| QYYZ |  |  |  |  |  |  | *** | 80.3 | 80.1 | 80.9 | 79.6 | 79.7 | 79.4 | 79.5 | QYYZ |  |  |  |  |
| NADC34 |  |  |  |  |  |  |  | *** | 96.2 | 84.9 | 85.4 | 85.2 | 85.4 | 86.2 | NADC34 |  |  |  |  |
| LNWK96 |  |  |  |  |  |  |  |  | *** | 84.7 | 85.4 | 85.2 | 84.6 | 85.2 | LNWK96 |  |  |  |  |
| JL580 |  |  |  |  |  |  |  |  |  | *** | 90.6 | 91 | 89.2 | 89.4 | JL580 |  |  |  |  |
| SD53-1603 |  |  |  |  |  |  |  |  |  |  | *** | 93.7 | 88.6 | 88.9 | SD53-1603 |  |  |  |  |
| NADC30 |  |  |  |  |  |  |  |  |  |  |  | *** | 88.7 | 89 | NADC30 |  |  |  |  |
| GDYS162 |  |  |  |  |  |  |  |  |  |  |  |  | *** | 95.4 | GDYS162 |  |  |  |  |
| GDHZ109 |  |  |  |  |  |  |  |  |  |  |  |  |  | *** | GDHZ109 |  |  |  |  |
|  | BJ-4 | VR2332 | CH-1a | HUN4 | JXA1 | GM2 | QYYZ | NADC34 | LNWK96 | JL580 | SD53-1603 | NADC30 | GDYS162 | GDHZ109 |  |  |  |  |  |
|  |  |  |  |  |  |  |  |  |  |  |  |  |  |  |  |  |  |  |  |
